# Membranous Nephropathy and Anti-Podocytes Antibodies: Implications for the Diagnostic Workup and Disease Management

**DOI:** 10.1155/2018/6281054

**Published:** 2018-01-08

**Authors:** Agnieszka Pozdzik, Isabelle Brochériou, Cristina David, Fahd Touzani, Jean Michel Goujon, Karl Martin Wissing

**Affiliations:** ^1^Nephrology Department, Centre Hospitalier Universitaire de Bruxelles (CHUB), Brugmann Hospital, Brussels, Belgium; ^2^Université Libre de Bruxelles (ULB), Brussels, Belgium; ^3^Pathology Department, AP-HP, Pitié Hospital, Paris, France; ^4^UPMC Université Paris 6, Paris, France; ^5^Centre National de Référence Maladies Rares: Amylose AL et Autres Maladies à Dépôts d'Immunoglobulines Monoclonales, Université de Poitiers, Poitiers, France; ^6^Pathology Department, Centre Hospitalier Universitaire de Poitiers, Poitiers, France; ^7^Nephrology Department, Universitair Ziekenhuis Brussel, Vrije Universiteit Brussel, Brussels, Belgium

## Abstract

The discovery of circulating antibodies specific for native podocyte antigens has transformed the diagnostic workup and greatly improved management of idiopathic membranous nephropathy (iMN). In addition, their identification has clearly characterized iMN as a largely autoimmune disorder. Anti-PLA2R1 antibodies are detected in approximately 70% to 80% and anti-THSD7A antibodies in only 2% of adult patients with iMN. The presence of anti-THSD7A antibodies is associated with increased risk of malignancy. The assessment of PLA2R1 and THSD7A antigen expression in glomerular immune deposits has a better sensitivity than measurement of the corresponding autoantibodies. Therefore, in the presence of circulating anti-podocytes autoantibodies and/or enhanced expression of PLA2R1 and THSD7A antigens MN should be considered as primary MN (pMN). Anti-PLA2R1 or anti-THSD7A autoantibodies have been proposed as biomarkers of autoimmune disease activity and their blood levels should be regularly monitored in pMN to evaluate disease activity and predict outcomes. We propose a revised clinical workup flow for patients with MN that recommends assessment of kidney biopsy for PLA2R1 and THSD7A antigen expression, screening for circulating anti-podocytes antibodies, and assessment for secondary causes, especially cancer, in patients with THSD7A antibodies. Persistence of anti-podocyte antibodies for 6 months or their increase in association with nephrotic proteinuria should lead to the introduction of immunosuppressive therapies. Recent data have reported the efficacy and safety of new specific therapies targeting B cells (anti-CD20 antibodies, inhibitors of proteasome) in pMN which should lead to an update of currently outdated treatment guidelines.

## 1. Introduction

After a short review of the pathophysiology of membranous nephropathy (MN), in the first part of this overview article, we describe the recently identified nephrotoxic antibodies and the corresponding antigens expressed by podocytes. They mainly consist of antibodies directed against neutral endopeptidase (NEP) in newborns [1], the cationic bovine serum albumin (BSA) [2] and aryl sulfatase in childhood [3], as well as the M-type phospholipase A2 receptor type 1 (PLA2R1) [4] and thrombospondin type 1 domain containing 7A (THSD7A) [5] in adults.

Antibodies against PLA2R1 are of particular clinical importance as they are detected in approximately 70% to 80% of adult cases of MN without apparent secondary causes, particularly in men [4, 6]. The prevalence of anti-PLA2R1 related MN appears to be lower (about 53%) in Japan than in other countries [7, 8]. Anti-THSD7A antibodies are detected in only 2% of adult patients with iMN with higher prevalence in women [5, 7]. The prevalence of anti-THSD7A related MN was 5.5% (7 out of 117 PLA2R1 negative MN cases) according to the immunoperoxidase staining detecting granular THSD7A antigen expression within extramembranous deposits [9] and 6.1% (4 out of 66) in a European cohort of PLA2R1 negative MN cases naïve of any immune therapy [5]. Antibodies against both PLA2R1 and THSD7A have been reported in about 1% of MN [9].

Both autoantibodies have been proposed as biomarkers of MN autoimmune activity [10–12]. High anti-PLA2R1 antibody levels have recently been reported as a reliable prognostic factor [13–16] which is likely to modify the indications for treatment to improve long-term outcomes of MN in the future.

More intensive screening for malignancy has been proposed in patients with THSD7A-related MN based on data from a cohort of 1276 patients with MN. Among these 8 women out of 40 with THSD7A-related MN developed cancer within 3 months from the diagnosis of MN [17].

## 2. Clinical Characteristics of Membranous Nephropathy

Membranous nephropathy (MN) is the most common cause of nephrotic syndrome in adults (≈25% of cases). The prevalence of MN varies widely by geographic regions and attains “epidemic” levels in China [10, 18]. Men are two times more likely to be affected by this disease than women [19]. MN occurs at any age, although it is rarely observed in children (10%) [20, 21]. The incidence of MN increases progressively with age, with a peak between 30 and 50 years [15, 19, 22–25].

MN is the 2nd or 3rd most common form of primary glomerulonephritis and evolves to end-stage renal diseases (ESRD) in 30% of patients [18]. In most cases, the onset of MN is not preceded by any prodromal manifestations such as signs of infection. Most patients present with a nephrotic syndrome: proteinuria above 3.0 g/24 h, hypoalbuminemia, edema, hyperlipidemia and lipiduria, and normal, or slightly altered, kidney function. The incidence of MN is probably underestimated given that the proteinuria below 2.0 g/day without a nephrotic syndrome has been described in 10% to 20% of cases [10]. Arterial hypertension has been reported in 10% to 55% of case series and is associated with a progressive decline of renal function [26, 27]. Acute renal vein thrombosis associated or not with pulmonary embolism has been rarely reported as the initial presentation of MN [28, 29].

Spontaneous complete remission of proteinuria is observed after a variable period of time (4 to 120 months) in approximately 30% to 40% of adult patients [30, 31]. In face of a sudden deterioration of the kidney function, tests should focus on the possibility of focal or diffuse lupus nephritis [32]. Primary MN may also be complicated by the development of focal and segmental glomerulosclerosis or by crescentic glomerulonephritis due to the development of anti-neutrophil cytoplasmic antibodies or antiglomerular basement antibodies [33].

## 3. The Clinical Forms of MN

Two forms of MN have classically been described: the idiopathic form and the secondary form of MN which represent 70% and 30% of cases, respectively [11, 12, 34].

Secondary MN is associated with the presence of immune complexes possibly containing foreign antigens and is most often observed in children as well as in patients over the age of 60. It occurs in the context of a number of infections including viral hepatitis B (HBV), hepatitis C, and hepatitis E or syphilis [35–37], certain autoimmune diseases (systemic lupus erythematosus, antineutrophil, Hashimoto's thyroiditis, and Sjögren's syndrome) [32, 33, 38], hyper-IgG4 syndrome [39], malignancies (digestive tract, lungs, and breast) [40], and the use of medicinal products (heavy metal salts such as gold salts in particular, D-penicillamine and its derivatives, nonsteroidal anti-inflammatory drugs, and levamisole-adulterated cocaine [41]) [41–44]. It has also been described in association with diseases such as sarcoidosis, in both adults [45–49] and children [50], sickle-cell anaemia, and graft-versus-host disease [51, 52].

Primary MN is related to the autoimmune disorders caused by the presence of circulating antibodies against native podocyte antigens NEP [1], PLA2R1 [4], and THSD7A [5] or by antibodies developed against external antigens such as cationic bovine serum albumin (BSA) [2] or aryl sulfatase [3]. Screening for these pathogenic antibodies therefore provides a new opportunity to more accurately define the etiology and the underlying pathways in the development of MN. The MN has been reported in Fabry disease [53]; however as far as we know the presence of anti-*α*-galactosidase A antibodies has not been documented.

## 4. Histological Features in MN

**Figure 1:**
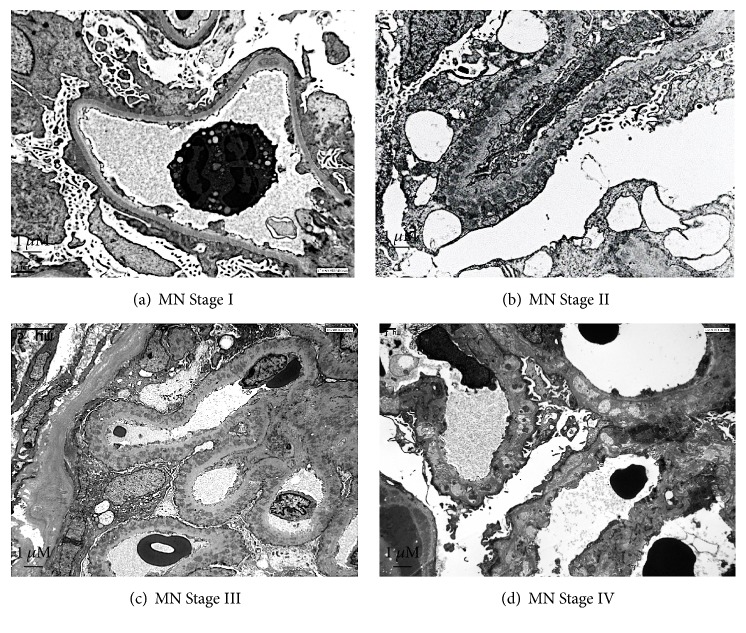
*Electron microscopy representative image of glomerular membranous nephropathy in adults*. (a) Stage I: electron dense deposits, irregularly distributed, at the outside of the glomerular basement membrane (GBM), without inflammatory reaction around the deposits. There is a variable degree of foot process effacement. (b) Stage II: spikes are irregular projections of the GBM among the subepithelial deposits. (c) Stage III: with progression of the disease, spikes become longer and incorporate the deposits in a thickened GBM. (d) Stage IV: the deposits lose their electron density until disappearance in the advanced stages of the process. Magnification ×3000. Kindly provided by Jean Michel Goujon, M.D., Ph.D. (Centre National de Référence Maladies Rares: Amylose AL et Autres Maladies à Dépôts d'Immunoglobulines Monoclonales, Université de Poitiers; Pathology Department, Centre Hospitalier Universitaire de Poitiers, Poitiers, France).

The key histological characteristic of MN is the formation of subepithelial (extramembranous) immune deposits, associated with variable degrees of alterations in the morphology of the glomerular basement membrane (“spikes”). The histology of MN depends on the timing of renal biopsy with respect to the onset of the disease and reflects the stage of disease [18]. Glomerular abnormalities, which are always diffuse, can be classified into 4 stages (Figure 1). Immunofluorescence is a useful technique in the diagnostic workup because it reveals the type and subtypes of immunoglobulin G within the deposits. Recent data suggest that the type of IgG subclasses in deposits provides important information on disease mechanisms and the potential for complement activation [54, 55]. All IgG subclasses have been observed in immune deposits. IgG1, IgG2, and IgG3 deposits are reported more frequently in secondary MN (lupus [56]; graft-versus host disease [57]; malignancies [58]). The IgG4 subclass has been most often observed in pMN and is in general absent in MN related to the malignancy [24, 58–60].

In a large cohort from Japan, specificity and sensitivity of IgG4 positive staining for detecting pMN corresponded to approximately 95% and 64%, respectively [7]. Glomerular IgG4 staining has been most frequently associated with PLA2R1 deposition in pMN and is rarely observed in HBV-related MN and in general absent in the membranous form of lupus nephritis [61]. The absence of glomerular IgG4 staining has been reported to be significantly more frequent in MN associated with malignancy than in pMN and has been identified as an independent predictor for occurrence of malignancy during the follow-up [59]. However, the data in the literature concerning the diagnostic value of IgG4 staining to differentiate between secondary MN due to malignancies and pMN is not completely clear-cut. In pMN related to PLA2R1 antibodies, IgG4 within extramembranous deposits is usually polytypic (involving several antibody specificities). On the contrary, monotypic IgG4 deposits have been reported in secondary forms of MN without anti-PLA2R1 antibodies in patients with hematological disorders [62] and lung neoplasia [63]. More intriguingly, the glomerular PLA2R1 and predominant/codominant IgG4 coexpression has also been reported in MN associated with malignancy [61]. These reports are therefore in contradiction with the initial observation that IgG4 and anti-PLAR1 antibodies are very rare or absent in patients with malignancy [59].

The presence of more than 8 inflammatory cells per glomerulus has been proposed to increase the likelihood of secondary MN related to the cancer, which should be thoroughly excluded by extensive investigation [64]. However, the sensitivity of this marker appears to be low because secondary forms of MN due to cancer have been reported without the presence of glomerular inflammatory cells [65].

## 5. Pathogenesis of Histological Lesions in MN: Glomerular Deposition of Immune Complexes, Complement Activation, and Tubulointerstitial Damage

As in other nephropathies, the persistence of high-grade albuminuria and/or proteinuria is associated with a progressive decline of glomerular filtration rate secondary to tubulointerstitial lesions (including tubular atrophy, interstitial inflammation, and fibrosis) in MN [66, 67]. While high-grade proteinuria is unequivocally a risk factor for the progression of renal dysfunction in general, in MN the situation might be more complex. The oxidative stress related to podocyte injury and glomerular sclerosis and the use of nephrotoxic drugs such as calcineurin inhibitors and renal vein thrombosis are among other potential mechanisms that might lead to the progressive decline of renal function [11]. An increase in levels of PLA2R1 antibodies has been reported as adverse risk factor for progression of renal failure especially in older patients. This risk was 2.8 times higher in men who presented progressive increase in total anti-PLA2R1 IgG levels as compared with women [68].

In addition, growing evidence demonstrates the role of complement activation in the progression of MN [69]. Immune complex deposits (Figure 1) in the space between the glomerular capillary basement membranes and podocytes contain (1) antigens which are either “foreign” or intrinsic to podocytes, (2) immunoglobulin subclasses directed against those antigens, and (3) components of the complement system, including in particular the membrane attack complex (MAC) C5b-9 whose effect on the podocyte cell membrane is responsible for cell injury and rearrangements of the glomerular basement membrane (GBM) which lead to proteinuria [11, 24, 70–73].

Complement activation probably also contributes to the pathogenesis and progression of renal insufficiency in patients with MN [71, 74, 75]. Indeed C5b-9 blocks podocyte autophagy by inhibition of lysosomal degradation of autophagosomes which increases podocyte apoptosis in human MN and cultured podocytes [76]. Inhibition of the podocyte autophagosomal/lysosomal system and ubiquitin proteasome system induces podocyte injury and worse proteinuria [77–79].

Moreover, tubulointerstitial injury (tubular atrophy and interstitial fibrosis) could be related to the exposure of tubular epithelial cells to various levels of protein in the tubular lumen and in particular ultrafiltrate-derived serum proteins containing complement factors (complementuria) [80]. The proximal tubular epithelial cells (PTEC) are of particular importance in the activation of urinary complement components. Indeed, the brush border on the apical membrane of PTEC bind properdin, which activates complement by the alternative pathway [81, 82]. It has been reported that, following the cleavage of C3, properdin may bind to serum C3b, stabilizes C3bBb convertase, and thus amplifies local activation of C3 and cell lesions [83]. The expression of properdin by PTEC has been reported in proteinuric diseases [82]. An* in vitro* study has demonstrated that exposure of PTEC to complement results in local C3-deposition and formation of MAC (C5b9) [84]. Therefore, PTEC could hypothetically be involved in the progression of MN by focal activation of the alternative complement pathway leading to progressive and sustained injury of PTEC.

## 6. The Podocyte Antigens and Pathogenic Antibodies

**Figure 2:**
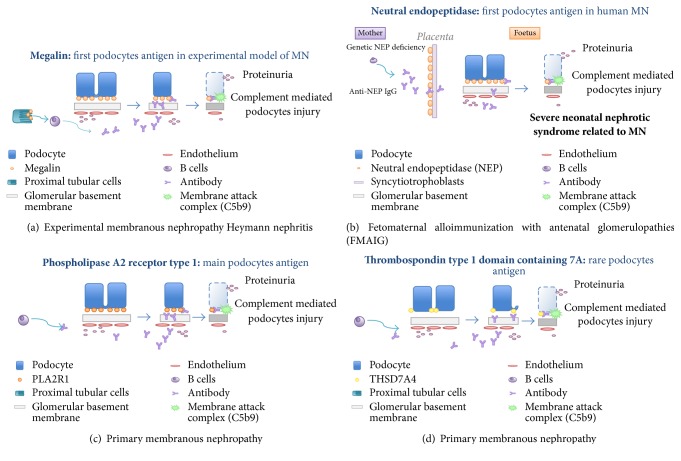
*Proposed mechanisms of experimental membranous nephropathy in rats (indirect alloimmunization) and human primary membranous nephropathies*.

The hypothesis of an autoimmune disease attacking podocytes was generated following observations in Heymann nephritis, an experimental model of MN in the rat (Figure 2(a)) [85]. In this model, megalin present on the surface of podocytes of rats has been identified as the target antigen responsible for the formation of extramembranous deposits.

Proof of the hypothesis that MN in humans also involves a podocyte antigen has been recently provided by two major discoveries (Figures 2(b)–2(d)). The first is the identification of neutral endopeptidase (NEP), an antigen involved in rare cases of neonatal MN [1]; the second is the characterization of antibodies directed against the PLA2R1 [4] and, more recently, against the THSD7A antigen [5].

The discovery of those autoantibodies targeting podocyte antigens has provided irrefutable proof that about 80% of MN previously considered as “idiopathic” are in fact caused by antibody-mediated autoimmune disease [4, 5, 86]. Accordingly the name “PLA2R1 or THSD7A related MN” needs to be used instead of iMN, which is no longer an appropriate terminology in the presence of corresponding autoantibodies [10–12]. The term iMN should therefore be restricted to the small minority of cases without specific autoantibodies and without an identifiable cause for secondary MN.

The diagnostic approach in pediatric forms of MN differs from adults and also requires the evaluation for (1) anti-NEP antibodies secondary to fetomaternal alloimmunization in newborns with MN and (2) deposition of specific antibodies against external antigens planted on podocytes such as cationic BSA in early childhood MN and aryl sulfatase in the case of MN in patients receiving enzyme substitution therapy [1–3].

### 6.1. Neutral Endopeptidase (NEP) and Fetomaternal Alloimmunization Related MN

Pierre Ronco and his team first identified a podocyte antigen responsible for MN in humans [1]. In this exceptional case of a neonate, MN was induced by the placental transfer of maternal antibodies directed against NEP in the foetal glomeruli during the last trimester of pregnancy [1]. NEP, also called CD10 or common acute lymphoblastic leukemia antigen (CALLA), is a metalloprotease involved in the degradation of peptides (enkephalin, natriuretic factors, endothelin, etc.) [87]. It is expressed by podocytes, the brush border of proximal tubular cells, as well as by endothelial cells, granulocytes, and syncytiotrophoblasts [88]. Mothers who are carriers of a homozygous or composite heterozygous mutation of the* MME* gene coding for NEP do not express the protein, which is no longer detected in the urine [89, 90]. Though the mothers have a deficiency of NEP, they are asymptomatic. During pregnancy, the immune system of these mothers is exposed for the first time to the NEP present on syncytiotrophoblasts. This leads to a fetomaternal alloimmunization process comparable to that associated with Rhesus incompatibility [91]. Anti-NEP alloantibodies of the IgG1 and IgG4 subclasses are detected in the maternal serum and breast milk, as well as transiently in the neonatal serum [89, 92]. The antigen has also been documented in the neonatal extramembranous glomerular deposits [89]. The responsibility of maternal anti-NEP antibodies in the pathophysiology of MN has been confirmed* in vivo* via the injection of maternal antibodies [93] into rabbits, which led to the development of nephrotic proteinuria and the formation of extramembranous deposits, characteristic of MN [1]. This work provided the first irrefutable proof of the involvement of a podocyte antigen serving as the target for pathogenic autoantibodies, thus confirming the pathophysiological mechanisms of experimental Heymann nephritis in humans [73]. Until today, 5 additional families have been identified with the previously reported* MME* gene truncating mutation [90].

### 6.2. Anti-Phospholipase A2 Receptor Type 1 Autoantibodies

The new pathophysiological concept of alloimmune MN has stimulated research aiming to identify human podocyte antigens that may act as targets for circulating pathogenic antibodies in humans, thus leading the way to the identification in 2009 of the first antigen responsible for primary MN in adults [4]. This publication was a cornerstone in the history of MN as it provided evidence that iMN in adults is actually an autoimmune disease associated with the production of anti-PLA2R1 antibodies and introduced a new terminology of PLA2R1 related MN [15, 23, 94–96].

Until now, the direct proof that human anti-PLA2R1 antibodies induce MN in experimental animal models has not been reported. Podocytes of neither rats nor mice express the PLA2R1 antigen. However, primary cultured podocytes derived from canine kidneys express PLA2R1* in vitro*, which offers new promises to demonstrate the direct pathogenicity of PLA2R1 antibodies in the future [97]. The induction of MN by monoclonal IgG3 kappa in recurrent MN is a potent argument for the direct pathogenic role of these antibodies in this disease [98].

### 6.3. Anti-PLA2R1 Antibodies, Genetic Risk Factors for Disease, and Immune Dominant Epitopes of PLA2R1

The involvement of genetic factors, in particular increased frequency of the HLA-DRw3, HLA-B8, and B18 alleles of the HLA histocompatibility system in patients with idiopathic MN, has been published as early as 1979 [99, 100]. A genomewide association study (GWAS) investigating over 280,000 individual polymorphic markers (*“single nucleotide polymorphism”* or SNP) found a highly significant association between MN and certain HLA-DQA1 polymorphisms [101]. The SNPs rs3749119, rs3749117, and rs4664308 in PLA2R1 and rs2187668 in HLA-DQA1 have been significantly associated with primary MN and anti-PLA2R1 antibodies [94]. Several reports have observed associations between PLA2R1 polymorphisms and the risk for development of primary MN [102], the response to immunosuppression (in association with HLA-DQA1 polymorphisms) [103], response to immunosuppressive therapy (a lower rate of remission for the C/G genotype at rs35771982) [104], and disease progression (C/T genotype at rs6757188) [104]. However, these observations remain to be independently confirmed [105].

Recently 2 independent groups have identified the region within the extracellular domain of the PLA2R1 antigen that is recognized by anti-PLA2R1 antibodies [106, 107]. The immunodominant epitope in PLA2R1 has been found in the terminal domain of PLA2R1 in particular in the cystine rich (CysR) region [107]. This region contains a conformational epitope recognized by 90% of human anti-PLA2R1 autoantibodies [106]. The exact mechanism leading to the modification of structure in CysR region of the PLA2R1 antigen remains unknown. However, the structural model gave rise to an interesting pathophysiological molecular mimicry hypothesis linking chronic bacterial infection and pMN [106]. It has been proposed that bacterial infection primes production of autoantibodies able to recognize the dominant epitope in PLA2R1 antigen on podocytes.

Moreover, an additional immune epitope within the PLA2R1 antigen associated with poor outcome of disease has been detected in the C-type lectin domains 1 and 7 (CTLD1 and CTLD7) [108]. The kinetic of immunization starts probably with the production of anti-PLA2R1 antibodies against the epitope in the CysR region, which is the most distal epitope, and is followed by immunization against epitope CTLD1 and finally CTLD7. This epitope spreading of antibody specificity from CysR, CysR + CTLD1 to CysR + CTLD1 + CTLD7 is associated with aging, increases in proteinuria, and poor outcome. On the contrary, in a small retrospective cohort of PLA2R1 related MN patients, the shift over time of anti-PLA2R1 antibody reactivity from a “broad” spectrum against CysR + CTLD1 + CTLD7 epitopes to the single CysR epitope was associated with better clinical outcome [109]. This observation raises the interesting perspective that treatment effects might be evaluated by the evolution of epitope spreading in addition to simple assessment of blood levels of circulating antibodies. However, this strategy has to be validated in prospective trials before implementation in routine clinical practice.

Recently, low levels of regulatory T cells and increases in circulating plasmablasts as well as in plasma cells have also been suggested as an additional new biomarker of MN activity and responsiveness to treatment [110–113].

### 6.4. Anti-Thrombospondin Type 1 Domain Containing 7 Autoantibodies

Recently, it has been reported that 15 out of 154 (10%) patients with PLA2R1-negative idiopathic MN had detectable anti-THSD7A autoantibodies. As for anti-PLA2R1 antibodies, the circulating serum anti-THSD7A antibodies were mainly IgG4 subtype but a weak presence of other subtypes has also been found [5].

Likewise PLA2R1 related MN, the prevalence of THSD7A related MN depends on ethnicity, material tested (serum or kidney biopsy), and technique used for the detection of circulating antibodies [5, 7, 9, 12, 17, 86, 114, 115]. Considering iMN patients, the prevalence of THSD7A related MN is more frequent (9.1%) in Japan [7] as compared with European cohort (2%, 4 out of 198 iMN patients) [115]. Along the same line, among 1276 patients with MN provided from combined cohorts (prospective and retrospective cohort from Hamburg and retrospective cohort from Boston) only 40 patients had detectable antibodies against THSD7A, indicating that THSD7A related MN is a very rare disease (3.1%) [17].

The prevalence of anti-THSD7A related MN among patients seronegative for anti-PLA2R1 antibodies appears to be higher in Asian countries (16% in China [116] and 19% in Japan [7]) as compared to those reported in the European and Boston cohorts (14% and 8%, resp.) [5].

In an intriguing paper from Japan, 8 patients with THSD7A related MN developed a malignancy within 3 months of follow-up. In this study, THSD7A related MN showed female predominance contrasting with the PLA2R1 related MN which is predominant in men [7].

The THSD7A related MN associated with malignancy has also been recently reported in European and Chinese patients [116, 117]. The possible pathological link between both diseases has been demonstrated by Hoxha et al. in a case of MN in a woman with gallbladder carcinoma [117]. Indeed, the expression of THSD7A antigen by tumor cells in the gallbladder and metastatic cells in lymph nodes suggested the hypothesis that anti-THSD7A antibodies were primed by malignancy secondary leading to the formation of immune complex within the subepithelial deposits in the glomerulus responsible for proteinuria. The chemotherapy resulted in the progressive decrease of circulating anti-THSD7A antibodies and concomitantly a decrease of proteinuria. Recently, highly variable expression of THSD7A with different staining patterns within different malignant cells types has been reported [118].

## 7. PLA2R1 and THSD7A Antigens Characteristics and Immunohistochemical Data

The PLA2R1 and THSD7A antigens are N-glycosylated proteins. PLA2R1 belongs to the superfamily of the lectin of type C receptors. It stimulates the endocyclic recycling involved in the clearance of soluble phospholipase A2. This enzyme is recognized as a potent inflammatory mediator [119, 120]. PLA2R1 is involved in cell senescence (apoptotic death) related to mitochondrial superoxide production [121].* In vitro*, soluble PLA2R1 receptor induces podocyte apoptosis through ERK1/2 and CPLA2 alpha signaling pathway [122].

The THSD7A is the most extensively characterized member of a family of extracellular matrix glycoproteins involved in the regulation of cellular behavior during tissue genesis and repair [123]. It interacts with glycosaminoglycans, calreticulin, and integrins regulating cellular adhesion in the extracellular environment. THSD7A mediates interaction of low-density lipoprotein receptor during its uptake and clearance at the surface of various cells and also regulates the interaction with fibrinogen during platelet aggregation.

The expression of PLA2R1 and THSD7A antigens is different in terms of localization, pattern, and intensity. In healthy controls the expression of PLA2R1 antigen is confined to the external side of the GBM with typically granular but relatively weak staining, while the THSD7A antigen is expressed within the GBM with a typical linear pattern and more intensely than PLA2R1 [9]. The expression of PLA2R1 is limited to podocytes in humans and not found in rodents [124].

The THSD7A antigen-staining pattern is very similar to that of nephrin. Under normal conditions in humans THSD7A expression is limited to the slit diaphragm, the podocyte's soma, endocytic compartment, and foot processes. THSD7A is found in rodents, but not on the GBM or on endothelial cells [125].

New heterologous mouse model of THSD7A related MN has been reported and confirmed the pathogenicity of THSD7A antibodies in the development of MN lesions [126].

Beck Jr. et al. using immunofluorescence method were first to demonstrate the presence of PLA2R1 antigen within extramembranous glomerular deposits [4]. The circulating anti-PLA2R1 antibodies have been detected by western blot in his work.

Commercial anti-PLA2R1 antibodies are used to identify the PLA2R1 antigen within extramembranous deposits in paraffin sections by an immunohistochemical technique [127]. Interestingly, this technique can be used retrospectively to diagnose PLA2R1 related MN in patients under immunosuppression who no longer have detectable circulating antibodies [47, 127]. This test can also be used to determine whether the PLA2R1 antigen is present in the native kidneys of candidates for renal transplantation, which is important to assess the risk of relapse [128]. Antigen detection within subepithelial deposits is more sensitive (86%) than detection of corresponding circulating antibodies (76%) at the time of kidney biopsy [129]. Positive staining for PLA2R1 in glomeruli strongly correlates with the presence of PLA2R1 antibodies in the serum [11].

## 8. Anti-PLA2R1 and Anti-THSD7A Antibodies and Primary MN

### 8.1. Specificity of Anti-PLA2R1 and Anti-THSD7A Antibodies in Primary MN

The discovery of anti-PLA2R1 and anti-THSD7A antibodies constitutes a major step forward in the management of patients with iMN, as both antibodies are very reliable diagnostic and prognostic markers of disease [10].

Anti-PLA2R1 antibodies are detected in approximately 70 to 80% of patients with iMN in Europe [130], the USA [4], and Asia [131], with the exception of Japan where the prevalence of these antibodies is lower than in other Asian countries (about 50% of patients with iMN) [8, 132].

The detection of anti-PLA2R1 antibodies in patients with nephrotic syndrome can be considered as a biomarker for the diagnosis of primary MN according to a recent meta-analysis (all study sensitivity 78% (95% CI: 66% to 87%) and specificity 99% (95% CI: 96% to 100%)) [6]. This important evidence can modify the indication for kidney biopsy in specific situations such as patients with a single kidney or those with increased risk of bleeding [14].

In addition to their significant sensitivity, the specificity of anti-PLA2R1 antibodies for MN has been proposed close to 100% as they have not been detected in healthy subjects and in non-MN glomerular diseases [4]. However, even if their prevalence is much lower, the presence of anti-PLA2R1 antibodies in secondary MN (active sarcoidosis, lupus nephritis, and HBV infection) has been reported [32, 33, 38–52]. However, the occurrence of anti-PLA2R1 autoantibodies in membranous lupus nephritis is very rare and they have never been observed in non-MN lupus nephritis. Recently, circulating anti-PLA2R1 antibodies have been reported in patients that had IgA nephropathy [133]. In retrospective study of 26 patients with biopsy-proven glomerular lesions that occurred in patient with sarcoidosis, MN preceded or occurred concomitantly with active sarcoidosis but was not reported in inactive sarcoidosis [46]. The expression of PLA2R1 antigen has been found in glomerular deposits in MN patients with sarcoidosis [45, 49]. Moreover, the PLA2R1 antigen within the glomerular deposits in 7.7% to 64% [61, 134] of a Chinese series of secondary MN related to HBV infection and was associated with presence of circulating anti-PLA2R1 antibodies [134].

Recent reports demonstrated that anti-THSD7A and anti-PLA2R1 MN are not mutually exclusive [9] as it has been previously proposed [5]. Described by Larsen et al. for the first time, double PLA2R1 and THSD7A MN is a very rare condition (0.7% of incident MN) [9, 116].

### 8.2. Anti-PLA2R1 and Anti-THSD7A Antibodies, Biomarkers of Disease Activity, Clinical Outcome, and Treatment Efficacy

Circulating anti-PLA2R1 antibodies reflect immunological activity of disease [4, 135] and have been shown to disappear before clinical remission of nephrotic syndrome [4] and to reappear in the circulation before clinical relapse [23].

Serum anti-PLA2R1 antibodies levels correlate with the degree of proteinuria and the reduction or resolution of anti-PLA2R1 antibodies levels has been shown to precede by at least 9 months of partial or complete remission proteinuria [135]. These data were confirmed by larger cohorts, which also found a correlation between anti-PLA2R1 antibodies levels and both the remission rate and the time to double serum creatinine [13, 15].

As compared to patients with non-PLA2R1 related MN, those with PLA2R1 related MN responded slower to immunosuppressive therapy [135, 136]. Moreover, patients presenting high titer of autoantibodies have more severe disease and a longer time to disease remission [137]. These observations illustrate the important role of anti-PLA2R1 antibodies levels as a prognostic marker of long-term clinical outcome of MN.

Similar data demonstrating that the disappearance of anti-THSD7A antibodies precedes remission of proteinuria and that an increase in the titer anti-THSD7A predicts the reappearance of proteinuria in a patient with a relapse has been reported in Chinese cohort [116].

### 8.3. Anti-PLA2R1 and Anti-THSD7A Antibodies, Biomarkers for Relapse of MN following Kidney Transplantation

To date, positive expression of PLA2R1 antigen has only been detected exceptionally in “de novo” cases as compared with recurrent MN in kidney transplant patients (8% versus 83%) [138, 139]. Nearly 50% of cases of recurrent MN on the kidney graft are associated with the presence of anti-PLA2R1 antibodies [128, 140, 141]. The detection or persistence of anti-PLA2R1 in kidney transplant patients has been associated with an increased risk of loss of graft function [12, 95, 139]. Recently, the recurrence of THSD7A related MN has been also reported and raises the issue of monitoring anti-THSD7A antibodies after renal transplantation [86]. However, it is actually unknown if relapses are more frequent in patients with related anti-THSD7A. Despite a broad consensus that assessment of anti-PLA2R1 in iMN and anti-THSD7A antibodies in anti-PLA2R1 serum negative iMN patients is indicated there is still no consensus on the frequency of assays during follow-up.

## 9. Anti-PLA2R1 and Anti-THSD7A Antibodies: Serological Detection Methods

**Figure 3:**
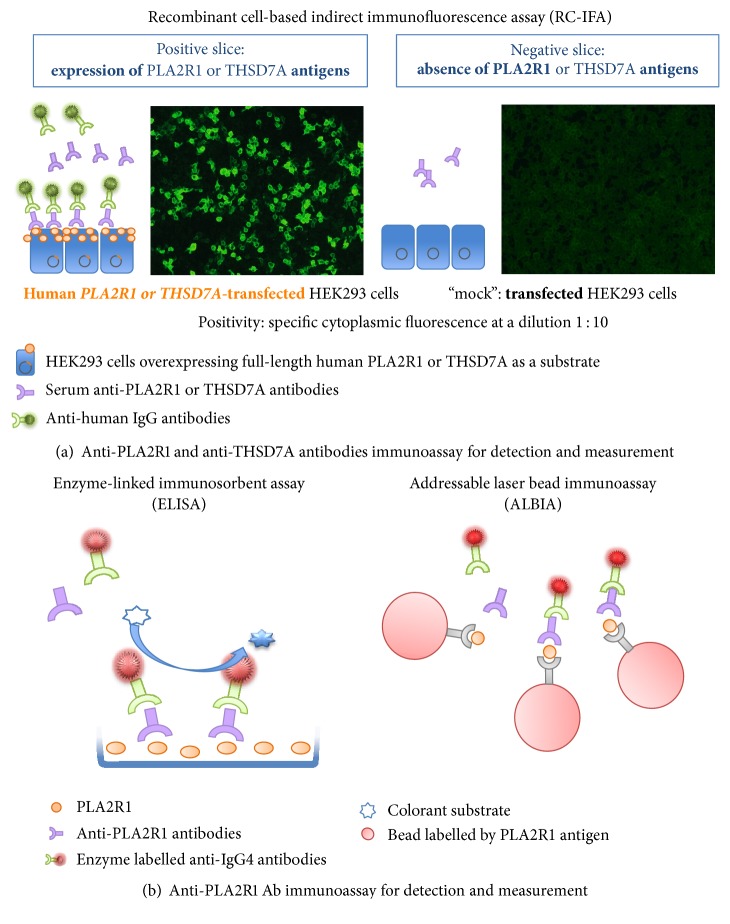
*Schematic presentations of available immunoassays detecting circulating anti-phospholipase 2 receptor 1 (PLA2R1) and anti-thrombospondin type 1 domain containing 7A (THSD7A) autoantibodies for diagnostic of primary membranous nephropathy (pMN)*. (a-b) Three standardized assays are currently available for diagnostic purposes of primary MN. The assessment of PLA2R1 could be performed using recombinant cell-based indirect immunofluorescence assay (RC-IFA), enzyme-linked immunosorbent assay (ELISA), or addressable laser bead immunoassay (ALBIA). The RC-IFA and ELISA are highly suitable for routine evaluation of pMN patients and are commercialized worldwide (Euroimmun AG, Luebeck, Germany). The ALBA developed by Mitogen Advanced Diagnostic Laboratory, Calgary, Canada, is a promising technique as it offers the possibility of analysing several antibodies in the same samples by one test. The RC-IFA uses the HEK293 human cell line overexpressing full-length human PLA2R1 protein. RC-IFA is a biochip format containing in one incubation field cells that express PLA2R1 antigen and control-transfected cells incapable of expressing PLA2R1. Using this test, anti-PLA2R1 antibodies are detected with very high specificity (nearly 100%) and high sensitivity (77%). Titers of anti-PLA2R1 antibodies decline during successful immunosuppressive therapy as well as during the spontaneous remission.

Techniques allowing for the detection and measurement of anti-PLA2R1 antibodies have developed very rapidly. Initial Western blots using protein extracts of human kidneys [4] or extracts of cells transfected with recombinant human cDNA for PLA2R1 have been replaced with a commercial bioassay using a recombinant cell-based indirect immunofluorescence assay (RC-IFA) technique [13] on slides containing “biochips” coated with human embryonic kidney cells (HEK 293) transfected with recombinant human cDNA PLA2R1 or THSD7A and nontransfected human embryonic kidney cells (HEK 293) (Figures 3(a) and 3(b)) [17]. The patient serum samples are incubated with increasing dilutions and the results are expressed as titers, similar to antinuclear antibodies. Comparison of the RC-IFA and western blots in a series of 42 cases revealed a very good qualitative correlation between the two tests (100%), with some quantitative discrepancies. Recently an immunoenzymatic method (ELISA) developed and is more quantitative than RC-IFA [13]. The addressable laser bead immunoassay (ALBIA) technique has been adapted for the detection of PLA2R1 antibodies and, in addition to its good diagnostic performance, ALBIA offers the benefit of multiplex analysis of other nephrotoxic antibodies and/or other immunological markers (Figure 3) [96]. A recent meta-analysis provided very high specificity and sensitivity of anti-PLA2R1 antibodies for the detection of pMN (99% and 78%, resp.) [6].

## 10. Other Potentially Pathogenic Antibodies

Antibodies against cytoplasmic alpha-enolase have long been known to be present in the serum of patients with primary and secondary MN (nearly 70%) [142] but never within the subepithelial deposits [143]. Antibodies against other cytoplasmic podocyte proteins, including aldose reductase and manganese superoxide dismutase 2, have also been detected in the serum and tissue eluates of microdissected glomeruli in biopsy samples from patients with MN [144]. These cytoplasmic antigens are not accessible to circulating antibodies under normal conditions. However, in the event of oxidative stress, they can migrate to the cell membrane and serve as targets for circulating antibodies. The activation of complement leading to the formation of MAC is a cause of oxidative stress in podocytes [71]. The initial lesions induced by the immune complexes involving PLA2R1 may induce oxidative stress responsible for this novel membrane expression of proteins, which are physiologically cytoplasmic, leading to the formation of new autoantibodies [10]. Their pathogenic role in the initiation and/or maintenance of the disease is still hypothetical.

## 11. Treatment of MN: Current Controversies

MN presents in a large spectrum of disease severity and has the potential for spontaneous remission without therapy and its progression is difficult to predict, which explains the therapeutic uncertainties that still persist today [145–147]. In less than 30% of the cases, the disease progresses slowly towards severe renal insufficiency despite optimal supportive care combined with classical immunosuppressive therapy [19, 22, 30, 147]. According to the KDIGO guidelines, the predictive factors for poor prognosis of MN are a decrease in glomerular filtration rate at the time of diagnosis, persistent nephrotic proteinuria at 6 months of optimal nephroprotection, male sex, age over 50 years, uncontrolled arterial hypertension, and the presence of interstitial fibrosis and tubular atrophy seen on a renal biopsy [148, 149].

### 11.1. Optimal Nephroprotection

Patients with nonnephrotic proteinuria (<3.0 g/day) without other symptoms such as increase in serum creatinine level or uncontrolled arterial hypertension are in general managed by optimal supportive care alone [147]. Optimal supportive care of MN consists of a standard renoprotective treatment (drugs that inhibit the renin-angiotensin-aldosterone system, control of arterial hypertension, dyslipidaemia, excess weight, and other cardiovascular risk factors) [148]. In a recent retrospective cohort study of patients with iMN in the absence of anti-PLA2R1 or anti-THSD7A antibodies and receiving optimal supportive care the 24-month outcomes were similar irrespective of the administration of immunosuppressive therapy [115], confirming previous reports of favorable evolution of some patients in the absence of immunosuppressive therapy [150].

### 11.2. Immunosuppressive Therapy

In 2017, the best therapeutic approach of MN still remains debated and varies between countries [151, 152]. Immunosuppressant therapies are in general administered to patients at risk of progression to the end-stage kidney disease (ESKD) based on persistent proteinuria for more than 6 months and/or impaired kidney function at the time of diagnosis of the disease [151–153]. Indeed, 86% renal survival at 10 years has been reported in a cohort of patients in whom immunosuppressive therapy based predominantly on cyclophosphamide and steroids was selectively administered because of bad renal prognostic [154]. Absence of remission or relapse following partial remission has been reported to be significantly associated with progression to stage 5 of chronic kidney disease [155]. Cytotoxic immunosuppressive treatment should be reserved for those forms with refractory nephrotic syndrome or renal insufficiency [154] as it is associated with a high rate of complications over the short term [neutropenia, anaemia, and thrombocytopenia] as well as the long term (neoplasms). Cyclophosphamide is preferred because of being less toxic than chlorambucil [156].

The efficacy of calcineurin inhibitors (cyclosporine and tacrolimus) used alone has been demonstrated by a large number of studies and confirmed by recent meta-analysis (2018 patients from 35 randomized controlled trials) [148, 156–160]. The high rate of remission of proteinuria contrasts the significant relapse rate after cessation of therapy (91% and 42%, resp.) [161]. In this study, 40% of patients with the highest proteinuria at 3-month follow-up and the highest time-averaged proteinuria were more likely to develop cyclosporine-induced acute kidney injury and to reach an adverse renal endpoint. In these patients switch to a regimen without calcineurin inhibitors has been proposed as a reasonable alternative.

Mycophenolate mofetil may be beneficial when associated with steroids [147]. MMF significantly reduced proteinuria but had no significant effect on the induction of complete remission and was also associated with an increased risk of relapse [162, 163].

Anti-CD20 antibodies [164] and adrenocorticotropic hormone (corticotropin) [165] offer new therapeutic opportunities that appear to be promising. A novel therapy based on proteasome inhibitor has been used with benefit in rituximab-resistant or partially responsive recurrent posttransplant membranous nephropathy [113].

The addition of the anti-CD20 antibody to the classical immunosuppressant treatment has been proposed to block the proliferation of B cells and the production of pathogenic antibodies in patients with recurrent MN [140, 166, 167].

Anti-PLA2R1 antibodies are valuable tools for the evaluation of treatment efficacy. Remission of nephrotic syndrome induced by RTX has been significantly associated with decrease in levels of circulating anti-PLA2R1 antibodies at 6 and 12 months [94] and after 24 months [135]. In the prospective randomized GEMRITUX trial, higher remission rate of proteinuria has been associated with decrease in circulating anti-PLA2R1 antibodies levels at 6 months after RTX [168]. Low anti-PLA2R1 antibodies level before treatment initiation or their absence and high albuminemia at 3 months were also significantly associated with remission in RTX treated patients. Adjunction of RTX to antiproteinuric standard therapy induced a higher remission rate of proteinuria after 6 months of randomization (35.1% versus 21%). The disappearance of circulating anti-PLA2R1 antibodies in 82% of patients preceded the increase of serum albumin level and the reduction in proteinuria [129] demonstrating the value of anti-PLA2R1 as a new helpful immunological biomarker for assessment of disease activity and treatment efficacy. Indeed, B cell-depleting strategies promote proteinuria reduction and clearance of serum anti-PLA2R1 autoantibodies [169–172]. Anti-CD20 monoclonal antibodies have been suggested as a first line of therapy for patients with iMN at risk of progression [112]. Indeed RTX is safe and noninferior to cyclosporine as long-term proteinuria remission has been reported in patients with iMN [173].

Retrospective analysis and prospective studies as well as randomized controlled trial have reported significant remission rate of nephrotic syndrome (65%) in patients with MN after RTX [129]. There is no consensus on the optimal regimen of RTX to treat MN and a variety of doses and administration frequencies have been used in different studies [174]. The safety profile has been confirmed in several studies; however the physician must be aware of rare but possible risk of acute nonischemic cardiomyopathy [175] and progressive urticarial dermatitis [176] related to the RTX administration.

Some authors suggest that depletion of peripheral B cells could be insufficient to ensure sustained remission; therefore higher doses and longer treatment duration could be considered when RTX is used to prevent the relapses [177, 178].

However, if more intensive treatment regimens have the potential to improve efficacy of RTX this has to be balanced against a higher risk of adverse effects and increased costs and should be evaluated by prospective trials. Waiting for the results from such clinical trials the B cell-driven protocol is proposed as the preferential regimen for RTX administration [174].

A new protocol based on the association of RTX and cyclosporine for 6 months, followed by a second cycle of RTX and tapering of cyclosporine during 18 months as a maintenance phase, demonstrated that proteinuria decreased by 80% at 6 months. Moreover complete remission has been reported in 54% at 12 months [179].

A novel therapy based on proteasome inhibitor (bortezomib, 4 doses of 1.3 mg/m^2^ over 2 weeks) efficiently reduced proteinuria in a case of recurrent MN after renal transplantation that had been only partially responsive to RTX [180].

In addition to rituximab, the development of new monoclonal antibodies targeting B cells such as ofatumumab and belimumab as well as new treatments such as bortezomib and eculizumab provides a variety of potential therapies with the potential to ultimately replace the nonspecific and toxic immunosuppressants that are the current standard of care of pMN [181]. In the near future treatment protocols for MN patients will probably be individualized based on the level of anti-PLA2R1 antibodies and proteinuria [182].

## 12. Actualized Workup of MN

**Figure 4:**
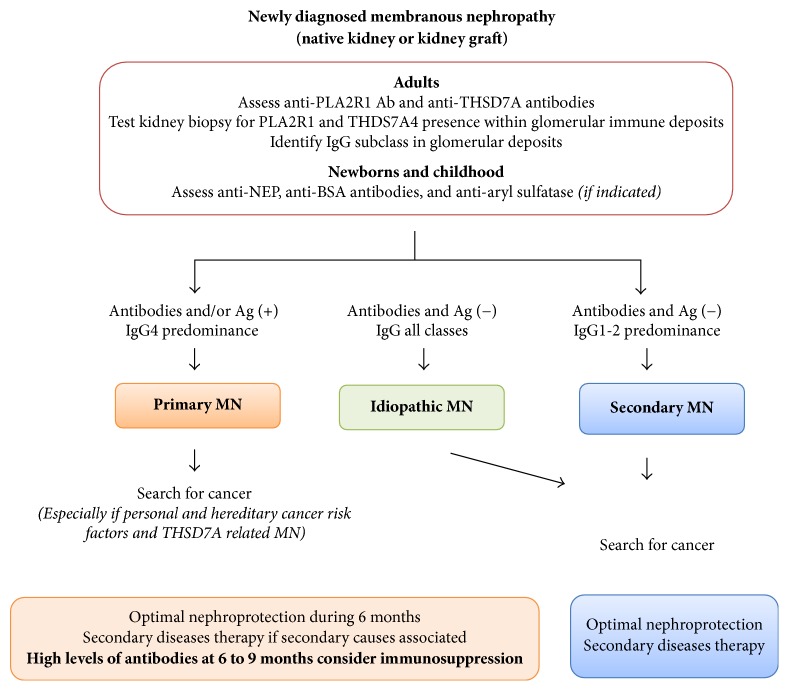
*Proposed summarized workup for diagnostic of membranous nephropathy in native kidney and kidney graft*. We propose a revised clinical workup for patients with MN. The screening for the presence of circulating antibodies against PLA2R1 and THSD7A, useful new tools reflecting that autoimmune activity is essential for differentiating “pure pMN” from secondary MN. It is only necessary to test for anti-THSD7A in those patients who have apparently primary MN and who are negative for anti-PLA2R antibodies. Moreover, the routine investigation of kidney tissue biopsy needs to include the histological evaluation of glomerular immune deposits for PLA2R1, cationic BSA in childhood, and aryl sulfatase in patients under enzymes replacement therapy. Testing for THSD7A antigen is only required in cases with suspected primary MN that is negative for PLA2R1 antigen or who are suspected of having cancer-related MN. Those histological biomarkers are far more specific for pMN than the presence of less than 8 inflammatory cells per glomerulus. Under these circumstances further etiological investigations could by stopped except in patients with important personal and hereditary risk factors for cancer and management focused on optimal nephroprotective and immunosuppressive therapy. Intensive screening for secondary causes especially for the presence of malignancies is still advised in patients with THSD7A related MN. On the contrary, the absence of circulating anti-PLA2R1 antibodies and/or PLA2R1 antigen in association with IgG1 or IgG2 subclasses within extramembranous deposits is associated with an increased likelihood of secondary MN related to cancer, which should be thoroughly excluded by extensive investigation. In this setting, the treatment of secondary causes and optimal nephroprotection without immunosuppressive therapy has been advised. The overlap of pMN and secondary MN, especially in sarcoidosis or HBV infection, is possible. It could suggest postponing immunosuppression in patients with HBV infection and focusing on antiviral therapy. In this situation, monitoring of anti-PLA2R1 antibodies could be helpful to assess renal response. The levels of circulating antibodies need to be regularly monitored during the follow-up to evaluate the long-term outcome of disease, efficacy of therapeutic interventions, and the risk of disease relapse or of recurrence in recipients of kidney transplants. Adjustments of interventions in function of the evolution of antibody levels are likely to have a central role in individualizing care of patients with pMN in the future. Persistence over 6 to 9 months or increase in antibodies level associated with nephrotic proteinuria is a strong argument in favor of adjunction of immunosuppression to optimal supportive care. It would be logical to start when antibody levels are high to prevent worsening proteinuria and, on the contrary, to stop or at least taper the immunosuppressive treatment once the anti-PLA2R1 antibody levels are no longer detectable, which occurs in general months before urine protein levels decrease.

A growing number of studies have generated strong evidence which calls for the adaptation of management of MN in the light of the present state of knowledge. The discoveries of anti-PLA2R1 anti-THSD7A antibodies literally shifted the paradigms in clinical workup of patients with MN, leading to the introduction of new terminology which is PLA2R1 or THSD7A related MN (Figure 4) [183]. High anti-PLA2R1 antibody levels have recently been reported as a new promising prognostic factor [13–16] and will probably modify the indications of treatment and how the disease is managed to improve the health and long-term outcomes of patients with MN. The present state of knowledge on anti-podocyte antibodies in the pathogenesis of MN and their role in monitoring disease activity and response to treatment is not reflected in international guidelines.

Awaiting the recommendations form revised KIDGO guidelines we propose a revised clinical workup for patient with MN. The previously proposed specificity of IgG4 expression within the deposits for pMN remains matter of debate. In contrast, the screening for the presence of circulating antibodies against PLA2R1 [4] and THSD7A [5], useful new tools reflecting autoimmune activity, is crucial to differentiating the “pure pMN” case from secondary MN. It is only necessary to test for anti-THSD7A in those patients who have apparently primary MN and who are negative for anti-PLA2R antibodies. Moreover, the routine investigation of kidney tissue biopsy needs to include the histological evaluation of glomerular immune deposits for PLA2R1 [53, 184], cationic BSA [2] in childhood, and aryl sulfatase in patients under enzymes replacement therapy [185]. Testing for THSD7A antigen is only needed in cases of suspected primary MN that is negative for PLA2R1 antigen or cases suspected of having cancer-related MN. Those histological biomarkers are far more specific for pMN than the presence of less than 8 inflammatory cells per glomerulus [65] (Figure 4) [3].

Under these circumstances further etiological investigations could be stopped except in patients with important personal and hereditary risk factors for cancer and management focused on optimal nephroprotective and immunosuppressive therapy [184]. Intensive screening for secondary causes especially for the presence of malignancies is still actually advised in patients with THSD7A related MN [17].

On the contrary, the absence of circulating anti-PLA2R1 antibodies and/or PLA2R1 antigen associated with predominantly IgG1 or IgG2 subclasses within extramembranous deposits is associated with an increased likelihood of secondary MN related to cancer, which should be thoroughly excluded by extensive investigation. In this setting, the treatment of secondary causes and optimal nephroprotection without immunosuppressive therapy has been advised [10, 148, 184].

The overlap of pMN and secondary MN, especially in sarcoidosis or HBV infection, is possible. Those observations could be simply interpreted as an unrelated coincidence of two diseases by some authors. An alternative hypothesis is that active sarcoidosis or chronic HBV infection prompts an immune response allowing the development of anti-PLA2R1 antibodies. This scenario has potential practical implications for the treatment strategy. It could suggest postponing immunosuppression in patients with HBV infection and focusing on antiviral therapy [35]. In this situation, monitoring of anti-PLA2R1 antibodies could be helpful to assess renal response.

The levels of circulating antibodies need to be regularly monitored during the follow-up to evaluate the long-term outcome of disease, efficacy of therapeutic interventions, and the risk of disease relapse or of recurrence in recipient of kidney transplants. Adjustments of interventions in function of the evolution of antibody levels is likely to have a central role in individualizing care of patients with pMN in the future.

Persistence over 6 to 9 months or increase in antibodies level associated with nephrotic proteinuria is strong argument in favor of adjunction of immunosuppression to optimal supportive care. Indeed, higher levels of circulating anti-PLA2R1 antibodies strongly correlate with the importance of proteinuria, predict a higher risk of recurrence of nephrotic syndrome and decline of renal function, and are associated with a lower rate and a longer time to obtain remission. These observations raise the question as to the best time to start and to discontinue immunosuppressive treatment. It would be logical to start when antibody levels are high to prevent worsening proteinuria and, on the contrary, to stop or at least taper the immunosuppressive treatment once the anti-PLA2R1 antibody levels are no longer detectable, which occurs in general months before urine protein levels decrease. Timely reduction of the intensity of immunosuppression should reduce the risk of overimmunosuppression and treatment-related side effects [168]. However, such a strategy of tailored immunosuppression remains to be validated in prospective studies.

In the near future nonspecific and toxic immunosuppressive treatments will undoubtedly be increasingly replaced by more specific and less toxic interventions directly targeting the key autoimmune disease mechanisms by targeting B cell proliferation and antibody production [182].

## 13. Conclusions

The recent discovery of an autoimmune mechanism related to anti-PLA2R1 and anti-THSD7A antibodies in adults and a fetomaternal alloimmunization against NEP in neonates has considerably improved our understanding of the pathogenesis of MN. Detection of anti-PLA2R1 and anti-THSD7A antibodies in serum and of the PLA2R1 and THSD7A antigens in subepithelial immune deposits constitutes a major breakthrough in the management of patients with MN.

Current research focuses on the pathophysiological mechanisms responsible for the production of these antibodies and their role in the process leading to podocyte lesions and interstitial renal fibrosis. Independently of the proteinuria, the anti-PLA2R1 and anti-THSD7A antibodies levels represent useful biomarkers for the diagnosis of MN and for monitoring the efficacy of therapeutic interventions. Approximately 50% of the cases of recurrent MN following kidney transplantation are associated with the presence of anti-PLA2R1 antibodies. However, the presence of these antibodies at the time of the transplantation is not constantly associated with a relapse. Anti-PLA2R1 epitope spreading emerged as new promising prognostic biomarkers for clinical outcomes. The systematic testing for a secondary cause including malignancy still needs to be performed, in particular in patients with THSD7A related MN.

We suggest that, after systematic screening for classical secondary cause of MN, the analysis of PLA2R1 and THDS7A (especially in MN associated with cancer) is indicated. Routine investigation should include screening for antibodies against PLA2R1 and THSD7A in anti-PLA2R1 seronegative MN and the corresponding target antigens in kidney tissue in adults, whereas deposits of NEP should be excluded in newborns as well as cationic BSA and aryl sulfatase during childhood. Despite the demonstration of efficacy and safety of new specific therapies targeting B cells (anti-CD20 antibodies, inhibitors of proteasome) in pMN, their place as first-line immunosuppression remains to be defined in treatment guidelines for MN that need to be updated in the light of the important evidence generated during the last few years. In the near future nonspecific and toxic immunosuppressive treatment will undoubtedly be progressively replaced by the immunosuppressive regimens incorporating selective inhibitors of B cell proliferation and antibody production.

## Additional Points

*Clinical Summary*. (i) The prevalence of anti-PLA2R1 and anti-THSD7A antibodies is approximately 70% and 2% in adults with idiopathic membranous nephropathy. (ii) Assessment of glomeral expression of PLA2R1 and of THSD7A antigen in case of PLA2R1 seronegative MN deposits is at present mandatory in idiopathic membranous nephropathy. (iii) The presence of anti-PLA2R1 antibodies has been reported in membranous nephropathy associated with secondary conditions (hepatitis B virus, membranous form of lupus nephritis, sarcoidosis, or malignancy); however their significance remains a matter of debate. (iv) The absence of anti-PLA2R1 in patients without immunosuppressive treatment and/or the absence of PLA2R1 expression within immune deposits should prompt systematic testing for a secondary cause of MN, including neoplasms, in particular in patients over the age of 60. Such a screening is also indicated in patients with anti-THSD7A related MN. (v) Circulating anti-PLA2R1 antibodies are considered valid biomarkers of immunological activity that correlate with markers of disease activity (degree of proteinuria) and are useful in predicting clinical outcomes such as response to treatment, long-term kidney function, and disease recurrence. (vi) Approximately 50% of cases of recurrent membranous nephropathy after kidney transplantation are associated with anti-PLA2R1 antibodies. (vii) Recently, epitope spreading within anti-PLA2R1 antibodies, levels of regulatory T cells, circulating plasmablasts, and plasma cells emerged as interesting additional tools for monitoring of immunological activity in primary membranous nephropathy. (viii) New heterologous mouse models of anti-THSD7A4 related MN are available at present. (ix) The monitoring of circulating anti-PLA2R1 and anti-THSD7A antibodies levels is indicated in patients with PLA2R1 or THSD7A-related MN, respectively. However, there is at present no consensus on the optimal monitoring schedule. (x) New specific therapies targeting B cells (anti-CD20 antibodies, inhibitors of proteasome) have demonstrated their efficacy and safety in primary membranous nephropathy, but their use as a first-line immunosuppression is still not recommended according to kidney disease improvement global outcome (KDIGO) 2012 guidelines that need to be revised.
